# Improved Intraoperative Visualization of Nerves through a Myelin-Binding Fluorophore and Dual-Mode Laparoscopic Imaging

**DOI:** 10.1371/journal.pone.0130276

**Published:** 2015-06-15

**Authors:** Victoria E. Cotero, Simon Y. Kimm, Tiberiu M. Siclovan, Rong Zhang, Evgenia M. Kim, Kazuhiro Matsumoto, Tatsuo Gondo, Peter T. Scardino, Siavash Yazdanfar, Vincent P. Laudone, Cristina A. Tan Hehir

**Affiliations:** 1 Diagnostics, Imaging and Biomedical Technologies, GE Global Research, Niskayuna, New York, United States of America; 2 Urology Service, Department of Surgery, Memorial Sloan—Kettering Cancer Center, New York, New York, United States of America; The Chinese University of Hong Kong, HONG KONG

## Abstract

The ability to visualize and spare nerves during surgery is critical for avoiding chronic morbidity, pain, and loss of function. Visualization of such critical anatomic structures is even more challenging during minimal access procedures because the small incisions limit visibility. In this study, we focus on improving imaging of nerves through the use of a new small molecule fluorophore, GE3126, used in conjunction with our dual-mode (color and fluorescence) laparoscopic imaging instrument. GE3126 has higher aqueous solubility, improved pharmacokinetics, and reduced non-specific adipose tissue fluorescence compared to previous myelin-binding fluorophores. Dosing and kinetics were initially optimized in mice. A non-clinical modified Irwin study in rats, performed to assess the potential of GE3126 to induce nervous system injuries, showed the absence of major adverse reactions. Real-time intraoperative imaging was performed in a porcine model. Compared to white light imaging, nerve visibility was enhanced under fluorescence guidance, especially for small diameter nerves obscured by fascia, blood vessels, or adipose tissue. In the porcine model, nerve visualization was observed rapidly, within 5 to 10 minutes post-intravenous injection and the nerve fluorescence signal was maintained for up to 80 minutes. The use of GE3126, coupled with practical implementation of an imaging instrument may be an important step forward in preventing nerve damage in the operating room.

## Introduction

Inadvertent nerve damage is one of the leading complications associated with many surgeries occurring in both open and minimal access surgical procedures. Some of the most commonly injured nerves include the peroneal division of the sciatic nerve during hip or knee replacement [1], saphenous or sural nerve during vein stripping [2], spinal accessory nerves during cervical lymph node dissection [3], brachial plexus injury during vascular bypass [4, 5], long thoracic nerve during mastectomy [6–8], phrenic nerve during coronary artery bypass graft [9–11] and cavernous nerves during radical prostatectomy [12, 13]. Complications arising from these injuries are related to the severity and location of the nerve injury and often result in symptoms that substantially reduce the patient's quality of life, such as loss of function and/or sensation, muscle atrophy, paralysis, and chronic neuropathy [14–16]. Cancer surgery requiring radical resection in the abdomen or pelvis is of particular importance, as many small nerves in those regions are involved in sensory, motor, and autonomic functions. In colorectal surgery and gynecologic surgery, preservation of autonomic nerves that control the bladder and bowel is of utmost importance after achieving cancer control. Nerve-sparing approaches have been developed, but they rely on white light optical identification of nerves with varied results [17]. In the field of urology, urinary and sexual dysfunction are common after radical prostatectomy as a result of nerve damage [18]. The cavernous nerves in the neurovascular bundle are often difficult to visualize during surgery not only because of anatomical variations in their location [19], but also their intricacy since they appear as more of a network of fine structures rather than a distinct anatomical structure [20].

The causes of nerve injury are varied but often results from limited ability to distinguish nerve fibers from the surrounding tissue; they can be mistaken for vessels, or they can be resected along with the targeted malignancy because of their proximity [21]. Current nerve-sparing techniques rely primarily upon anatomic landmark identification, which is highly dependent on the surgeon's experience, or intraoperative electrical stimulation devices, which have not proved sufficiently precise and sometimes detect nerve damage after it has occurred [13].

Intraoperative identification of some critical structures, such as vasculature and lymph nodes, has been successfully demonstrated using fluorescence image-guided surgery in preclinical and clinical studies [22–24]. Optical contrast agents are coupled with optical imaging instrument to provide visualization and increased contrast of otherwise indistinguishable anatomic features during surgery [25–29]. While the small incisions afforded by minimal access surgery bring benefits, such as shorter hospital stays, the limited field of view makes visualization of anatomic structures even more challenging than in open surgery. Such procedures are directed by imaging through a camera, which lends itself to enhanced visualization of critical structures such as nerves.

We have previously reported on two nerve selective bis-styryl fluorophores, GE3082 and GE3111, which upon systemic injection effectively cross both the blood-nerve and blood-brain barriers producing fluorescence in myelinated nerves [30–33]. The lipophilic nature of these fluorophores requires a specialized intravenous (IV) formulation to enhance and maintain aqueous solubility; with the optimal nerve-to-muscle contrast ratio typically achieved around 4 h post-injection in rodents. Furthermore, the extent of deposition into surrounding adipose tissue seen with these previous contrast agents causes a decrease in the ease of visualization of labeled nerves. In the current work, we report on a newly synthesized fluorophore, GE3126, coupled with our custom dual-mode laparoscopic imaging system [34]. This system allows for color and fluorescence imaging with a single camera using time-multiplexed detection and synchronized illumination.

Here we report the dosing and kinetics of GE3126 in murine and porcine models. GE3126 has substantially improved pharmacokinetics and reduced non-specific adipose tissue fluorescence intensity, enhancing nerve visibility. In anticipation of future clinical translation, a preliminary functional observation (Irwin screening) and supplemental battery tests (coordination, startle, analgesia and locomotion) in rodents were used to determine central and peripheral nervous system effects followed by real-time imaging in a porcine model.

## Materials and Methods

### Synthesis of GE3126 (2-(4-(4-(4-(4-Aminostyryl)-3-methoxystyryl)phenylsulfonyl)piperazin-1-yl)ethanol hydrochloride)

**Fig 1:**
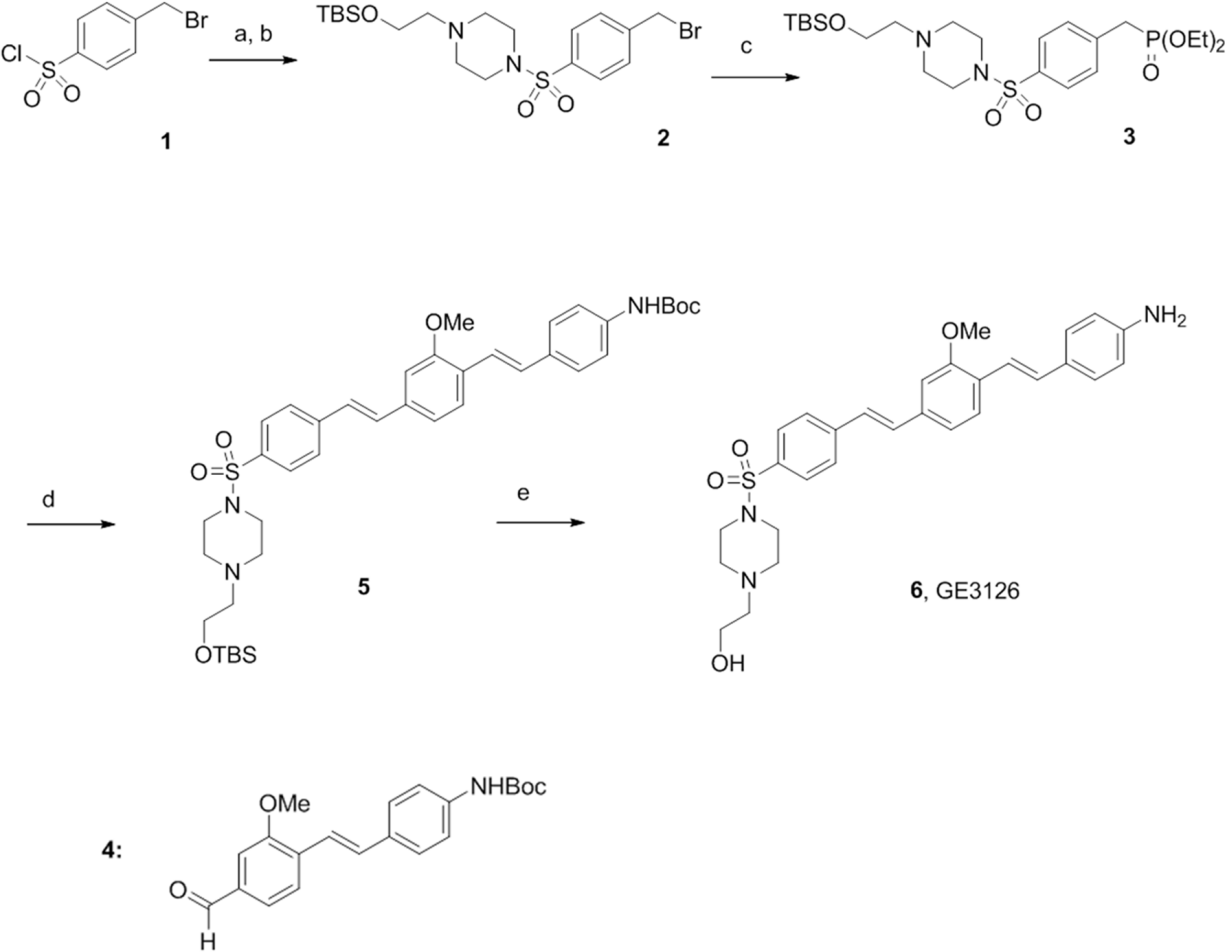
Schematic chemical synthesis of GE3126 (details are in S1 File). The legends a-e are abbreviated experimental conditions that correspond to each step in the synthesis. (a) 2-hydroxyethylpiperazine, diisopropylethylamine (DIPEA), CH_2_Cl_2_; (b) *tert*-butyldimethylsilylchloride (TBDMSCI), room temperature, overnight; (c) P(OEt)_3_, 100°C, 78 h; (d) **4**, potassium tert-butoxide (tBuOK), THF, 60°C, 12 h; (e) 4N hydrochloric acid, dioxane, then aqueous NaHCO_3_, CH_2_Cl_2_.

Compound GE3126 was synthesized in a stepwise procedure as illustrated in Fig 1. Reaction of sulfonyl chloride **1** with N-hydroxyethylpiperazine followed by addition of t-butyldimethylsilyl chloride produced the protected sulfonamide benzylic bromide **2** in 47% yield over two steps. The Arbuzov rearrangement upon addition of triethyl phosphite proceeded to give phosphonate **3.** The reaction proceeded cleanly and the starting material and product were readily separable by medium pressure liquid chromatography (MPLC). Horner-Wittig olefination with the previously reported aldehyde **4** [31] produced compound **5** in 41% yield following MPLC purification. Treatment with 4N hydrochloric acid in dioxane followed by solvent exchange and neutralization gave compound **6** (GE3126) in 95% yield and better than 96% purity by nuclear magnetic resonance (NMR) spectroscopy. The presence of fluorescent impurities was determined using high performance liquid chromatography (HPLC) with attached fluorescence detection with excitation at 375 nm, and emission at 470 nm. Removal of traces of fluorescent impurities was achieved through a final purification by reversed phase HPLC, eluting with water-acetonitrile gradient at 0%-70%. The final purification provided >99.9% optical purity. The fluorophore was stable as a free base, and was stored at -20°C under dry nitrogen. Details of the synthetic methodology can be found in S1 File.

### Physical and Optical Properties of GE3126

Methods for spectroscopic and physicochemical characterizations were as previously described [31]. Briefly, absorbance spectra were taken using a Lambda 20 UV/Vis spectrometer (Perkin Elmer, Waltham, MA). The wavelength of maximum absorbance was then used as the excitation wavelength for the collection of the fluorescence emission spectra on a steady state fluorimeter (Photon Technology International, Birmingham, NJ). A 10 mM stock solution of GE3126 in dimethylsulfoxide (DMSO) was prepared and was used in the preparation of 10 μM and 1 μM solutions of GE3126 in various solvents for the absorbance and fluorescence measurements, respectively. The partition coefficient, logD of GE3126 at pH 7.4, was calculated using Accelrys Discovery software (San Diego, CA). To compare its solubility with previously reported fluorophores, GE3082 and GE3111 [31], an excess of GE3126 was dissolved in a formulation containing 58.5% distilled water, 30% 2-hydroxypropyl-β-cyclodextrin, 10% propylene glycol, 1% polyethylene glycol-300, and 0.5% DMSO. The dissolution was followed by centrifugation (10 min at 12,000 g). The supernatant was diluted 1,000-fold in DMSO and its absorbance spectrum was taken. The maximum soluble concentration was calculated using Beer-Lambert’s equation with the extinction coefficient equal to 32,200 M^-1^cm^-1^.

### *In Vivo* Fluorescence Imaging

#### Imaging Instrument

Initial *in vivo* imaging dosing and kinetics studies were performed using a fluorescence stereomicroscope (SteREO Lumar V12, Carl Zeiss Inc., Thornwood, NY) equipped with a multispectral imaging camera (Nuance, CRI, Woburn, MA) as previously described [31], and using an exposure time of 5 s. Excitation of GE3126 was achieved using a filter centered at 406 nm with a 15 nm bandwidth. The fluorescence emission spectrum was recorded at wavelengths ranging from 420 to 720 nm at 10-nm increments using the attached multispectral camera. Numerical data presented herein represent the area under the curve for wavelengths ranging from 550 to 720 nm, which mimics our previous study using a 550 long pass filter [32]. This range also included the fluorescence emission maxima for nerve, muscle and adipose tissue.

Real-time intraoperative imaging was achieved with a dual-mode laparoscopic fluorescence imaging instrument previously described [34]. The instrument has a compact 90 g, 658x492 pixel GigE color camera (acA640-90gc Basler AG, Ahrensburg, Germany), and 405-nm blocking filter (BLP01-405R Semrock, Rochester, NY), mounted on the distal end with a standard 10-mm zero-degree surgical laparoscope with a 70-degree field of view (Linvatec T1000; Conmed Linvatec, Largo, FL). Although the instrument is sufficiently sensitive to visualize the contrast agent at or near peak absorption [34], the instrument sensitivity must be maximized to capture kinetics of uptake and clearance intraoperatively. Therefore, to increase irradiance, the light guide was removed and the illumination source was directed onto the field, while maintaining laparoscopic visualization. For processing of porcine model imaging data, individual frames extracted from real-time video were analyzed using ImageJ, an image-processing program developed at the National Institutes of Health. From these images, the fluorescence intensity was measured for manually-selected regions each corresponding to nerves, muscle and adipose tissue. The average fluorescence represented the average intensity from three regions of interest for each tissue type.

#### Formulation of GE3126 for Intravenous Administration

GE3126 was prepared for IV administration by dissolving in a buffer containing 0%–15% propylene glycol (P355-1; Thermo Fisher Scientific, Waltham, MA), 0%–20% (2-hydroxypropyl)-β-cyclodextrin (2-HPβCD) (Sigma H5784; Sigma-Aldrich, Saint Louis, MO), and 65%–85% distilled water (Sigma W3500). The IV formulation was brought to a final pH of 7.4 using 1M of hydrochloric acid. Complete solubility of the agent in the formulation mixture was verified using (1) visual observation for particulates, (2) centrifugation (5 min at 12,000 g) followed by observation, (3) dissolution in a physiologically relevant buffer (e.g., Sorenson's phosphate buffer) followed by visual observation and UV/Vis analysis, and (4) assessment of sedimentation and particle size using dynamic light scattering, as previously described [31]. The final formulation for IV injection contained 80% distilled water, 10% 2-HPβCD (w/v), and 10% propylene glycol (v/v).

#### Animals

All procedures on rodents were approved by the Institutional Animal Care and Use Committee (IACUC) at GE Global Research (Animal Care and Use Protocol 2010-11-NL, 2012-12-NL, NL2014-03, and 2014–04. Male CD- 1 mice ranging in body weight from 25 to 30 g, and Sprague-Dawley rats ranging in body weight from 250 to 300 g, were purchased from Charles River Laboratories (Wilmington, MA) and housed at 22– 23°C on a 12-h light/dark cycle. Mice were maintained on Prolab RMH3500 mouse chow (LabDiet, Framingham, MA) and water ad libitum, while rats were maintained on LabDiet 5001 rodent food (LabDiet) and water ad libitum. On the day of the experiment, mice and rats were anesthetized using 2%– 4% isofluorane and given a single tail vein injection of either GE3126 in IV formulation buffer or IV formulation buffer alone (control animal). The animals were then returned to the home cage until the designated time-point for imaging. Euthanasia was performed with an IV injection of pentobarbital (100 mg/kg body weight).

The Memorial Sloan- Kettering Cancer Center IACUC approved the Animal Use Protocol for the pigs used this study (Protocol ID 3174). All procedures were conducted in accordance with the Guide for the Care and Use of Laboratory Animals [35]. Two Yorkshire pigs weighing 30 to 35 kg (Archer Farms, Darlington, MD) received food and water ad libitum and were housed for a minimum of 7 days for quarantine, acclimation, and observation prior to the procedure. A fentanyl patch (25 μg/h) was placed 12– 14 h prior to surgery. The night before surgery, the pigs fasted for 12 h but were allowed water. All pigs were sedated with tiletamine/zolazepam (4.4 mg/kg), given glycopyrrolate (0.007 mg/kg), and a local infiltrate of bupivacaine (0.25%) was delivered subcutaneously along the midline prior to laparotomy. Each pig was intubated and maintained on isoflurane (1.5%–2.5%) during the surgery. A 16-gauge angiocatheter was placed in an ear vein for blood collection and drug administration. Normosol-R was given at a rate of 10–15 mL/kg/h during the procedure. Continuous monitoring of vital signs, temperature, and oxygenation was performed to assure the pigs’ safety and health. Each pig was euthanized at the conclusion of the imaging study with an IV injection (1 mL/4.5 kg, concentration of 390 mg/mL) of Euthasol (Virbac AH, Fort Worth, TX).

#### Dosing and Kinetics

The kinetics and dose-response for GE3126 were determined in adult male CD-1 mice. Three mice were evaluated for each dose and each time point. The kinetics study was performed prior to detailed investigation of the dosing to determine the optimal time window for imaging of GE3126. Each mouse received a single injection of 16 mg/kg of GE3126, and then euthanized at different time points (0.5, 1, 2, 3 and 4 h post IV injection), dissected and imaged.

Following the kinetics study, mice were given a single dose of GE3126 1 h prior to imaging of key designated nerves. Doses of GE3126 in this study ranged from 1.6 mg/kg to a maximum concentration of 46.6 mg/kg. Control mice were given a single injection of IV formulation (vehicle only) and imaged to determine background fluorescence.

#### Modified Irwin Screening and Supplemental Battery Tests

A preliminary safety assessment of GE3126 was conducted using a functional observation battery (Irwin screening) with additional supplement tests on naïve wild-type rats [36]. Each of four male Sprague-Dawley rats received a single IV injection of 35 mg/kg GE3126 formulated as follows: 80% sterile purified water, 10% propylene glycol, and 10% 2-HPβCD. To exclude the effects of the excipient, four rats were injected with the formulation buffer alone. The following physiologic criteria, described below, were monitored at 0, 15, 30, 60, 120, 240, 360 and 480 minutes post injection. A final assessment was done 24-hours post injection followed by tissue collection of key nerves for *ex vivo* analysis.

**In home cage observation:** Presentation of piloerection, tremor/convulsion, ptosis, exophthalmia, mydriasis, sedation, scratching, head tilt, straub tail or observed changes in respiration, vocalization, posture, salivation, lacrimation and excitation.

**Open area observation:** Presentation of catalepsy, akinesia, abnormal gait or jumping, head twitch/tilt, fore paw treading, stereotypies or loss of coordination.

**Handheld observation:** Observed changes in startle response, aggression, tactile sensitivity, pinna reflex, pupillary reflex, corneal reflex, abdominal tone, twitch response, grasping changes, loss of righting reflex, pain/analgesia, respiration, hydration, cyanosis and body temperature.

Results of the modified Irwin screening were used to determine the potential for nervous system injury resulting from an acute dose of GE3126.

#### Intraoperative Visualization Using the Dual-mode Laparoscope

Male Sprague-Dawley rats were given a single tail vein injection of 12.2 mg/kg GE3126 in IV formulation buffer (80% distilled water, 10% 2-HPβCD, and 10% propylene glycol), or formulation buffer alone without GE3126 (control animal). After 1 h, the rats were euthanized using compressed CO_2_ gas and prepared for imaging under both open (brachial plexus) and minimal access (closed thoracic cavity) procedures. For the porcine model, 27 mg of GE3126 was solubilized in 10 mL of IV formulation buffer (80% distilled water, 10% 2-HPβCD, and 10% propylene glycol) as described above, and was given into a Yorkshire pig as a bolus injection to a final concentration of 0.74 mg/kg GE3126. Incisions were made into the left brachial plexus and video was recorded just prior to GE3126 injection (time = 0), for the first 5 min post-injection, and then every 10 min until 100 min. Nerves in the retroperitoneum were also visualized starting at 90 min. Subsequent to imaging, the pig was euthanized as described above, and imaged tissues were completely resected for fluorescence microscopy and pathologic analysis. Tissues were fixed in 10% neutral buffered formalin for 72 h, paraffin embedded, and sectioned to 4 μm thickness and affixed to slides stained with hematoxylin and eosin (H&E). A board-certified veterinary pathologist with 13 years of experience performed histologic assessments.

## Results

### *In vitro* Properties of GE3126

Fluorophore GE3126 was synthesized and purified to >96% purity by NMR spectroscopy and >99.9% optical purity. The molecule was prepared based on the chemical scheme shown in Fig 1, in both milligram and gram-scale quantities (details of which are in S1 File).

GE3126 has a molecular weight of 519.7 g/mole and a logD value at pH 7.4 of 3.5 (Table 1). Its logD was significantly lower than GE3111 (4.5) and GE3082 (5.0) [31], indicating a molecule with decreased non-specific partitioning in adipose tissue. To directly compare its aqueous solubility with that of GE3111 and GE3082, GE3126 was formulated in the same manner as those fluorophores (as previously described) [31]. The decrease in lipophilicity resulted in a significant increase in aqueous solubility of GE3126 to 82.7 mM when dissolved in 58.5% distilled water, 30% 2- HPβCD, 10% propylene glycol, 1% polyethylene glycol-300, and 0.5% DMSO. This solubility value was 1.6 times and 9.6 times greater than GE3111 and GE3082, respectively under identical formulation buffer composition.

**Table 1:** *In vitro* properties of the fluorophores.

Compound	LogD at pH 7.4	Aqueous solubility (mM)^a^
GE3126	3.5	82.7
GE3111^b^	4.5	51.5
GE3082^b^	5.0	8.6

^a^ Measured maximal solubility when dissolved in 58.5% distilled water, 30% 2- HPβCD, 10% propylene glycol, 1% polyethylene glycol-300, and 0.5% DMSO

^b^ From Reference [31]

The optical properties of GE3126 are dependent on the local solvent environment (Table 2). A bathochromic shift, which increased with solvent polarity, was observed in the absorbance and fluorescence emission maxima, which were in the visible region. In the more polar solvents, the Stokes shifts were greater than 200 nm.

**Table 2:** Optical properties of GE3126 in different solvents.

Optical properties	Ɛ(M^-1^cm^-1^)	Abs Max (nm)	Em Max (nm)	QY (%)
DMSO	32,200	414	621	9.6
Ethanol	35,560	400	610	9.1
IV formulation	32,284	395	583	15.1
Olive Oil	7,900	395	476	80.1

Ɛ = molar extinction coefficient; Abs Max = absorbance maximum wavelength; Em Max = emission maximum wavelength; QY = quantum yield.

In olive oil, which is a highly non-polar solvent that mimics the fatty acid content of adipose tissue [37], its maximal fluorescence emission underwent a hypsochromic shift of greater than 120 nm compared to the more polar solvent, DMSO. A significant increase in quantum yield in olive oil (80.1%) was observed compared to DMSO (9.6%).

### Preclinical Rodent Fluorescence Imaging

Initial *in vivo* imaging of peripheral nerves was performed using the commercial multispectral fluorescence stereomicroscope. Following optimization of dosing and kinetics, real-time intraoperative visualization was performed using the custom dual-mode laparoscopic system. The final formulation buffer for IV injection consisted of 80% distilled/deionized water, 10% 2- HPβCD, and 10% propylene glycol.

**Fig 2:**
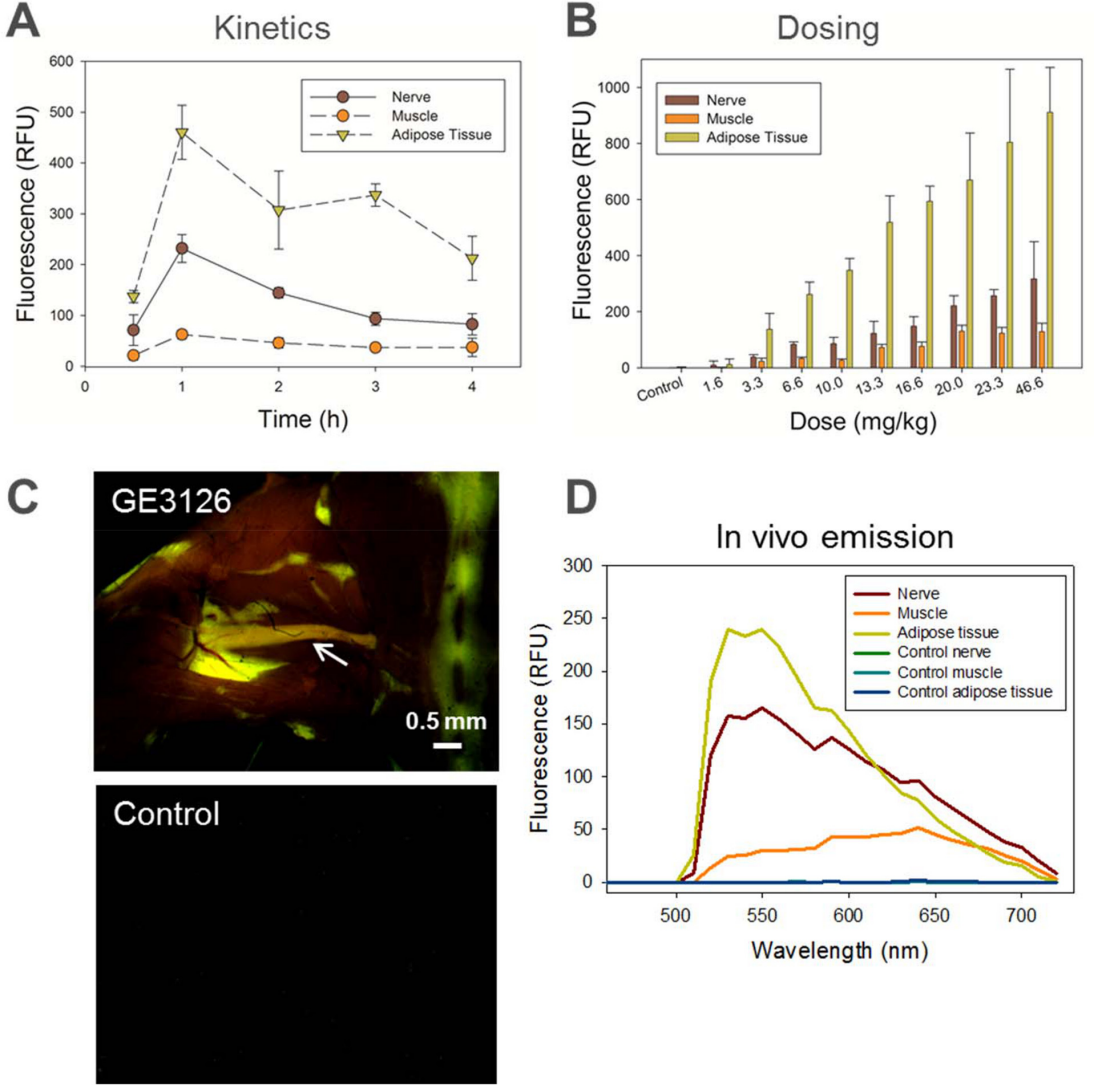
Kinetics and dosing in mice following IV administration of GE3126 formulated with 80% distilled/deionized water, 10% 2- HPβCD, and 10% propylene glycol. Imaging was performed using a commercial fluorescence stereomicroscope with multispectral detection. Excitation was achieved using a filter centered at 406 nm with a 15 nm bandwidth. Numerical data represented the area under the curve for emission wavelengths ranging from 550 nm to 720 nm acquired in sciatic nerves, adjacent muscle and adipose tissue, with n = 3 mice per group. (A) For kinetics, each mouse was given 16 mg/kg of GE3126 and imaging was performed at 0.5, 1, 2, 3, or 4 h post-injection. (B) In the dosing study, mice were given 1.6, 3.3, 6.6., 10.0, 13.3, 16.6, 20.0, 23.3, or 46.6 mg/kg of GE3126 and imaging was performed 1 h post-injection. Control mice were given a single injection of IV formulation (vehicle only) and imaged to determine background fluorescence. (C) Representative fluorescence multispectral image of a mouse injected with 16.6 mg/kg of GE3126. The sciatic nerve (reddish-orange) is indicated by an arrow. Note fluorescence labeling of adjacent adipose tissue in green. The control animal tissue appeared dark. Scale bar ~0.5 mm. (D) Emission spectra of nerve, muscle, and adipose tissue are shown to illustrate spectral separations between tissue types in both GE3126-treated mouse and control mouse.

We first evaluated the kinetics of uptake and clearance of GE3126 in mice. A dose of 16.6 mg/kg of GE3126 was used for measuring the nerve fluorescence at 0.5, 1, 2, 3 and 4 h after IV injection. Maximum sciatic nerve and adipose tissue fluorescence were observed at 1 h post injection and decreased rapidly through subsequent time points (Fig 2A). At this time point, the adipose tissue fluorescence was about two times higher in intensity than the nerve fluorescence. Low fluorescence signal was seen in muscle across all time points, with the best nerve-to-muscle ratio observed at 1 h post-injection, with a value of 3.7. The nerve-to-adipose tissue ratio was highest at 0.5 and 1 h post injection, with a value of 0.5. No animal was used for more than one time point to prevent introduction of confounding data (resulting from anesthetic or surgical interruption) that could alter *in vivo* pharmacokinetics and pharmacodynamics.

Based on the kinetics study, an imaging time point of 1 h post-injection was used for GE3126 in concentrations ranging from 1.6 to 46.6 mg/kg formulated in 80% distilled deionized water, 10% 2-HPβCD and 10% propylene glycol. One hour after injection, the mice were euthanized and key nerves were exposed. Fluorescence emission intensity increased in the sciatic nerve in a dose-dependent manner (Fig 2B). The increase in nerve fluorescence became more modest as the dose increased above 16.6 mg/kg, suggesting saturation of signal or saturation of binding of the fluorophore to the myelin sheath. A slower saturation in signal was observed in adipose tissue as the concentration of GE3126 increased, while minimal fluorescence was seen in adjacent muscle tissue. The control mouse was given a single IV injection of formulation buffer only for the purpose of measuring autofluorescence under identical imaging conditions. In this case, the autofluorescence for nerve, muscle and adipose tissue were less than 10 relative fluorescence units (RFU).

Fig 2C shows a representative example of fluorescence imaging in a mouse taken using the multispectral fluorescence stereomicroscope. Under a multispectral camera, the surrounding muscle tissue was dark, and the color of the fluorescence image of exposed sciatic nerve appeared red-shifted compared to the adjacent adipose tissue. The spectral differences of GE3126 between the nerve and adipose tissue environments were evident in the corresponding *in vivo* spectral emission curve in Fig 2D. Between 625–750 nm, the nerve appeared to have higher fluorescence intensity than the adipose tissue. The control mouse tissue was completely dark under identical imaging conditions and the corresponding fluorescence intensities for control nerve, muscle, and adipose tissue were below 5 RFU (Fig 2C and 2D).

### Laparoscopic Imaging in Rodents

**Fig 3:**
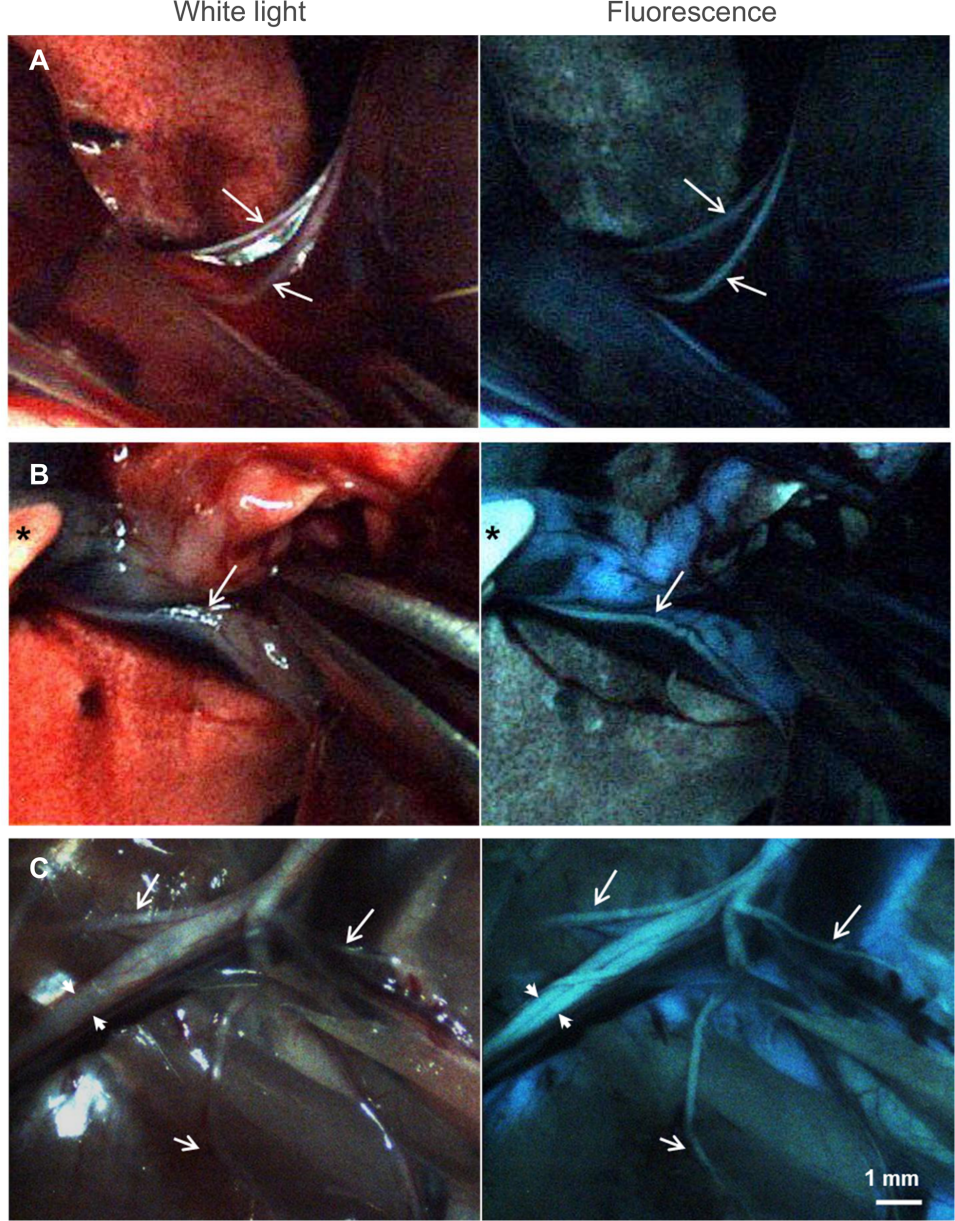
Individual frames extracted from real-time video recorded during minimal access (A, B) and open (C) surgery of a rat injected with 12.2 mg/kg of GE3126 using a custom dual-mode laparoscopic imaging instrument. Arrows are pointing to nerves. A small incision was made in the diaphragm allowing access of the laparoscope to the thoracic cavity highlighting the areas around the phrenic nerve (A) and the vagus nerve (B). White light images (left column) identify anatomical markers (such as lung and blood vessels), and fluorescence images (right column) show small diameter nerves easily obscured by surrounding fascia. (C) Open incision in the brachial plexus. The medial and lateral cords (arrowheads) are more easily distinguished in fluorescence mode. Smaller nerves that are more visible under fluorescence, such as the suprascapular, anterior thoracic, and thoracodorsal nerves are shown with white arrows. Asterisk in (B) is a gloved finger of the operator. Scale bar ~1 mm.

We then performed fluorescence imaging in rats using the dual-mode laparoscopic instrument built in-house. A small incision was made near the diaphragm allowing access of the laparoscope to the closed thoracic cavity of the rat. Video was recorded and individual frames for both white light and fluorescence channels were extracted showing the phrenic nerve obscured by attached fascia (Fig 3A) and the vagus nerve obscured by a mix of blood vessel and adipose tissue (Fig 3B). In both cases, fluorescence showed the presence and location of the small diameter nerves more clearly than white light imaging alone. Imaging in an open incision was also performed to expose the larger nerves of the rat brachial plexus (Fig 3C). Under white light, the medial and lateral cords (arrow heads) appeared as one bundle. Under fluorescence visualization, the medial and lateral cord along with some of the smaller nerves appeared more distinct and noticeable.

### Modified Irwin Screening

**Fig 4:**
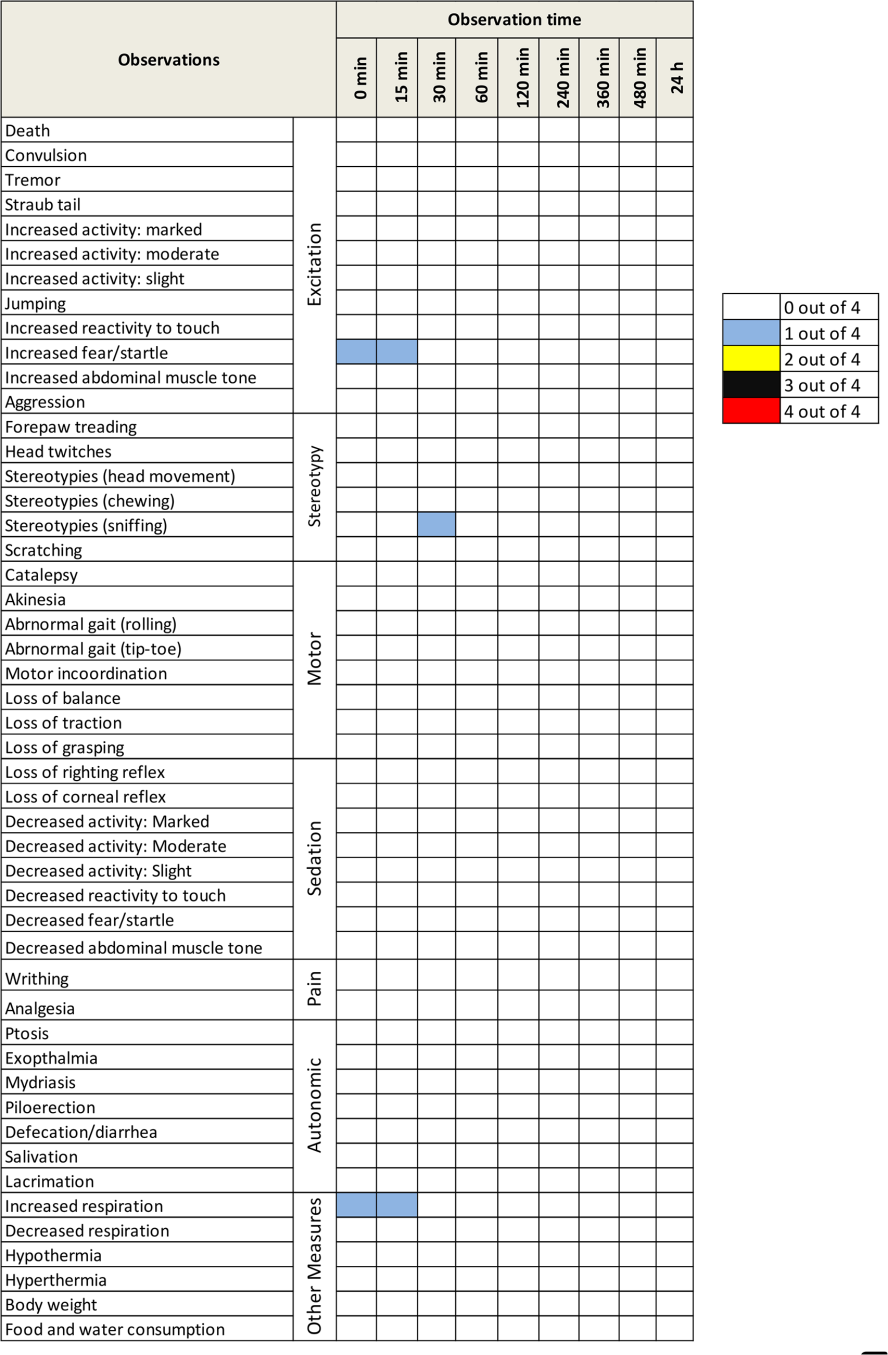
Modified Irwin screening and supplemental battery tests in rats to assess potential adverse effects on the nervous system. Four Sprague-Dawley rats were injected with 35 mg/kg GE3126 and observed at defined time intervals, up to 24 h post-injection for gross signs of reactions. The number of rats (of the 4 injected showing a reaction was reported for each adverse effect. Note that the same rat exhibited the transient effect in respiration, startle and sniffing. Four control rats (not shown) were injected with formulation buffer alone and none exhibited any reactions.

This fluorophore and its predecessors (GE3082 and GE3111) bind to myelin, with a strong affinity for myelin basic protein [30]. Because myelin is a major constituent of the brain, we sought to determine any potential adverse effects on the central nervous system. The modified Irwin screen was used for a preliminary evaluation of the potential of the fluorophore to induce neurotoxic effects in the central and peripheral nervous system, as determined through observation of physiologic, behavioral, autonomic and motor changes in the rodent [38]. A single high dose (35 mg/kg) of GE3126 was injected into four rats, while 4 rats were injected with the formulation buffer alone to serve as control. Each rat was observed at defined intervals up to 24 h after IV-injection. Observation environments included home cage and open area assessment, external stimulation assessment (handling) and basic physiologic exam (Fig 4). For each animal, all behavioral findings were entered manually into Microsoft Excel, with a score of 1 indicating a change in the observed parameter and a score of 0 indicating no change in observed parameter from baseline. No overt behavioral change was observed in animals receiving a single injection of formulation buffer alone (no GE3126) at any time point (Fig 4). Before dosing (0 min) and at 15 min after dosing of 35 mg/kg GE3126, 1 out of 4 rodents showed an increase in startle and elevation in respiratory rate. This was no longer present 30 min after dosing and was not thought to be of pharmacological relevance. No observable change in Irwin parameters were seen in 3 of the 4 rats injected with GE3126, before dosing or up to 24 hours post injection.

No significant effect on the central or peripheral nervous system was seen with a single injection of GE3126 at 35 mg/kg in male Sprague-Dawley rats. Furthermore, no visible change in organ morphology was seen in any animal receiving either a single injection of GE3126 or vehicle. Additional electromyogram measurements will be performed to confirm the lack of direct nerve damage resulting from binding of GE3126 to the myelin sheath of the nerve. However, for preclinical purposes the findings from the modified Irwin studies suggest a lack of neurotoxicity with acute administration of a high dose of GE3126.

### Intraoperative Imaging in Porcine Model Using the Dual Mode Laparoscope Instrument

**Fig 5:**
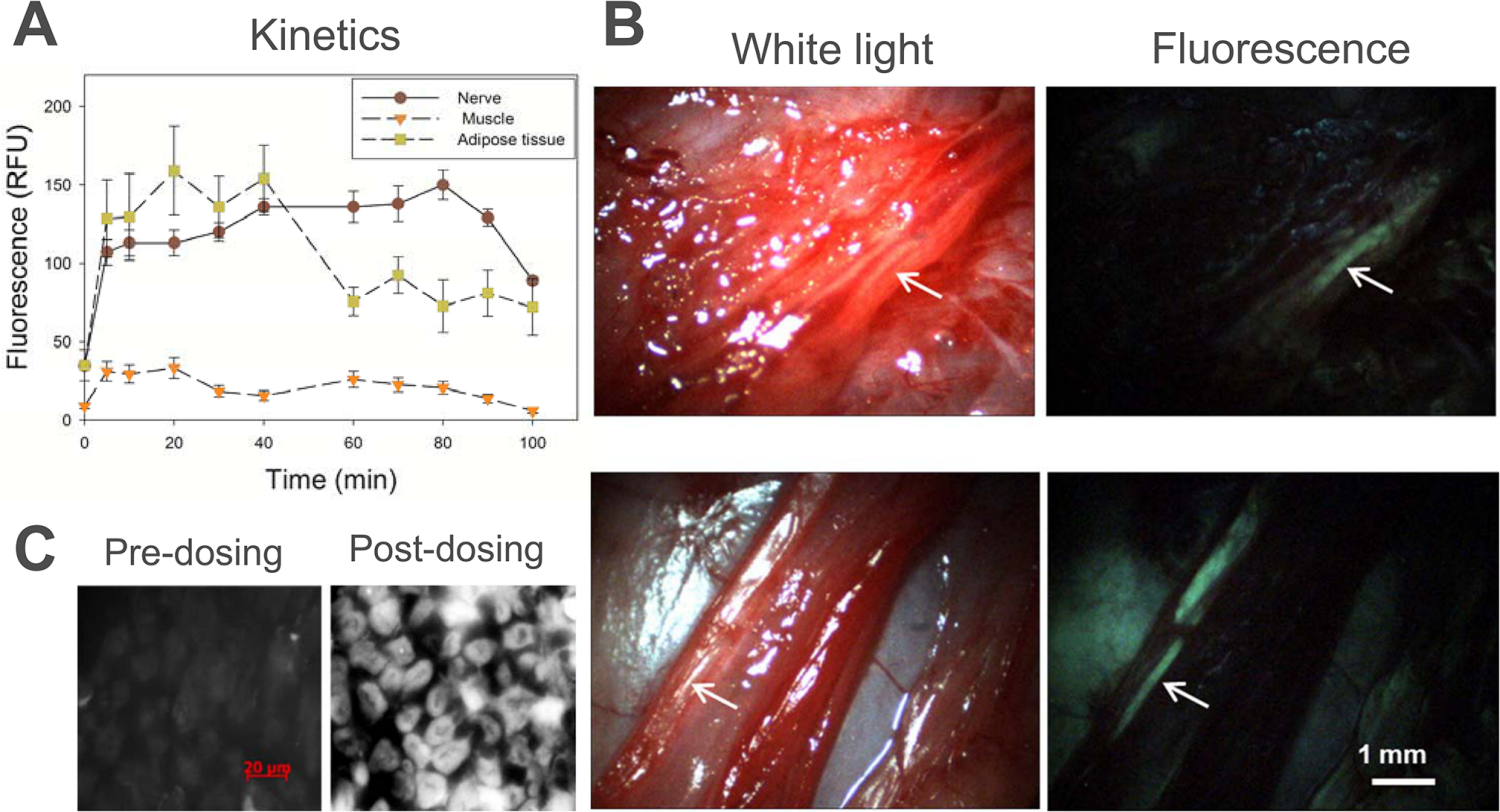
Dual-mode laparoscopic imaging in the porcine model. A dose of 0.74 mg/kg GE3126 was injected into the pig. An incision was made into the left brachial plexus and video was recorded just prior to GE3126 injection and at defined time points up to 100 min post-injection. (A) Individual frames were extracted and the fluorescence intensities of nerve, adjacent muscle and adipose tissue were plotted over time. (B) Representative white light and fluorescence images extracted from the video show the brachial plexus (top) and the retroperitoneal region (bottom) at ~80 min post-injection. Nerves are shown with white arrows. (C) Fluorescence microscopy images of a pig nerve tissue section taken prior to injection of GE3126, and at ~90 min post-injection. Scale bar in B ~1 mm; scale bar in C = 20 μm.

Once we established that GE3126 did not exhibit major adverse effects in rats, and we had optimized the fluorescence imaging conditions in rodents, we carried out surgeon-directed intraoperative nerve imaging studies in a porcine model. The rodent dose of 12.2 mg/kg was scaled to match the porcine model by body surface area. A final dose of 0.74 mg/kg GE3126 was administered by IV injection into the pig. The pig’s vital signs remained normal throughout the procedure. The uptake of the fluorophore in nerve was rapid, and nerve fluorescence was observed as early as 5 min after IV-injection (Fig 5A). The surrounding muscle tissue was dark at all times. The adipose tissue fluorescence was also observed within 5 min post injection. Surprisingly, the contrast between nerve and surrounding muscle tissue was much improved in the pig compared to the rodents. The range of nerve-to-muscle ratio from 5 min to 90 min post-injection was 3.4–9.3. Moreover, the fluorescence intensity of the fluorophore in adipose tissue was much less in pig compared to rodent. At the worst, the nerve-to-adipose ratio was 0.8 in the porcine model, compared to a ratio of 0.3–0.5 in mice (Fig 2A and 2B). Nerve fluorescence in pig was maintained up to 80 min post-injection, and clearance from the nerve was observed subsequently. Adipose tissue fluorescence was lower than nerve fluorescence after 40 min.

**Fig 6:**
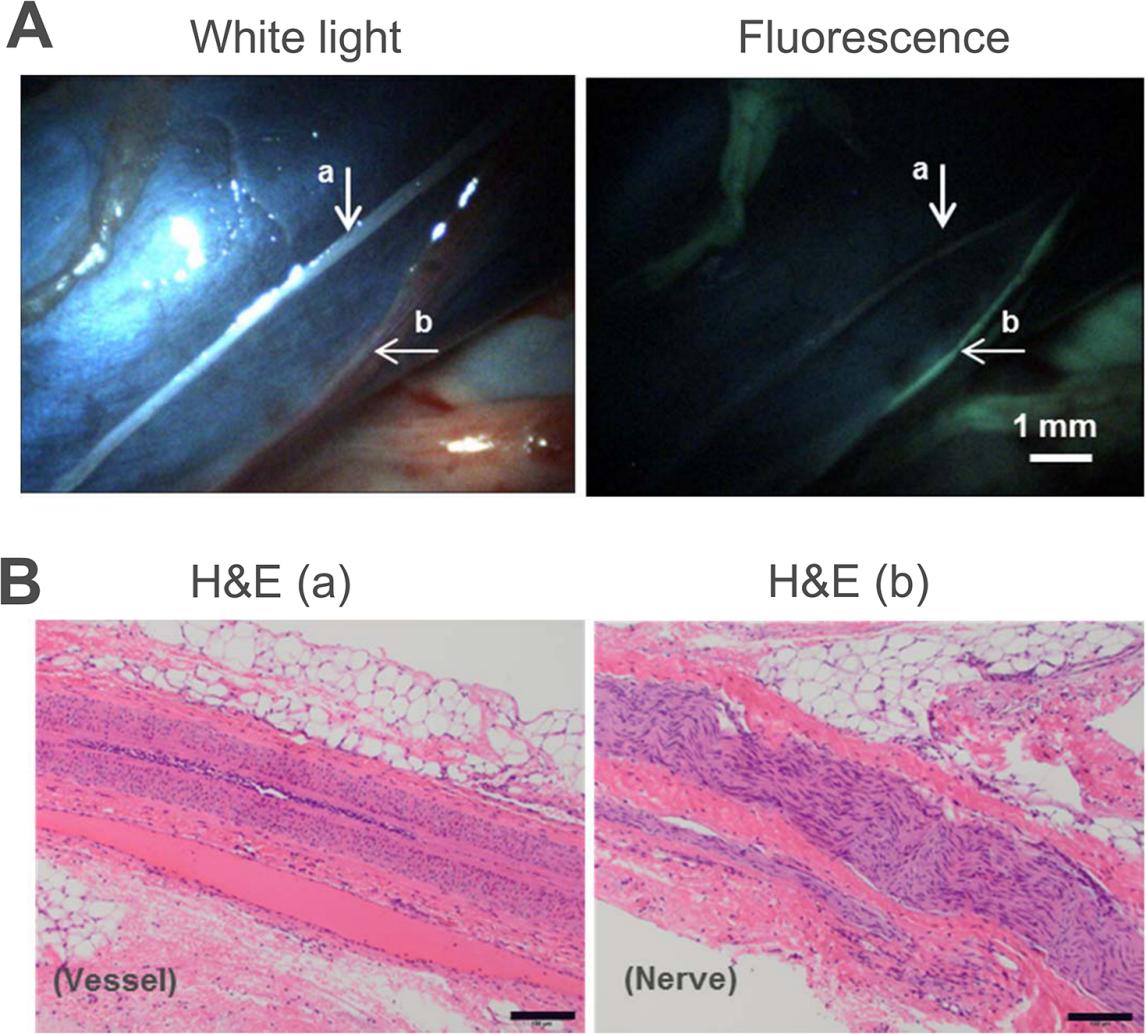
Dual-mode laparoscopic imaging in a porcine model and corresponding histology confirmation. (A) White light imaging (top left) showing a vessel, marked “a”, and a harder-to-visualize nerve, in the vena cava, marked “b”. The corresponding fluorescence image (top right) shows the fluorescently labeled nerve “b” more clearly than white light imaging. (B) H&E staining of tissue sections of “a” and “b” confirmed the identity of the non-labeled blood vessel (left) and labeled nerve (right). Scale bar in A~ 1 mm; scale bar in B = 100 μm.

Figs 5B and 6A show representative examples of the raw, unmodified white light and corresponding fluorescence images of the brachial plexus as well as nerves in the retroperitoneal area. The visualized nerves appeared green and ranged in diameter from 250 to 400 microns. The muscle area was dark and fluorescence from adjacent adipose tissue was less intense. A cross section of a resected brachial nerve showed that the characteristic donut-shaped myelin sheaths surrounding the axons were fluorescent by microscopic visualization (Fig 5C).

To confirm the identity of the fluorescently labeled nerve structure, a portion of the labeled structure (labeled “b” in Fig 6A) was resected for microscopic confirmation by H&E staining by a pathologist. As a control, an adjacent non-labeled vessel-structure was also resected for comparison (labeled “a” in Fig 6A). H&E staining of a longitudinal section revealed that the fluorescently labeled structure “b” was a myelinated nerve fascicle, showing the distinguishing wavy appearance. The dense nuclei belonging to Schwann cells and endoneurial cells within the fascicle were discernible. The non-labeled structure “a” was confirmed to be a blood vessel.

**Fig 7:**
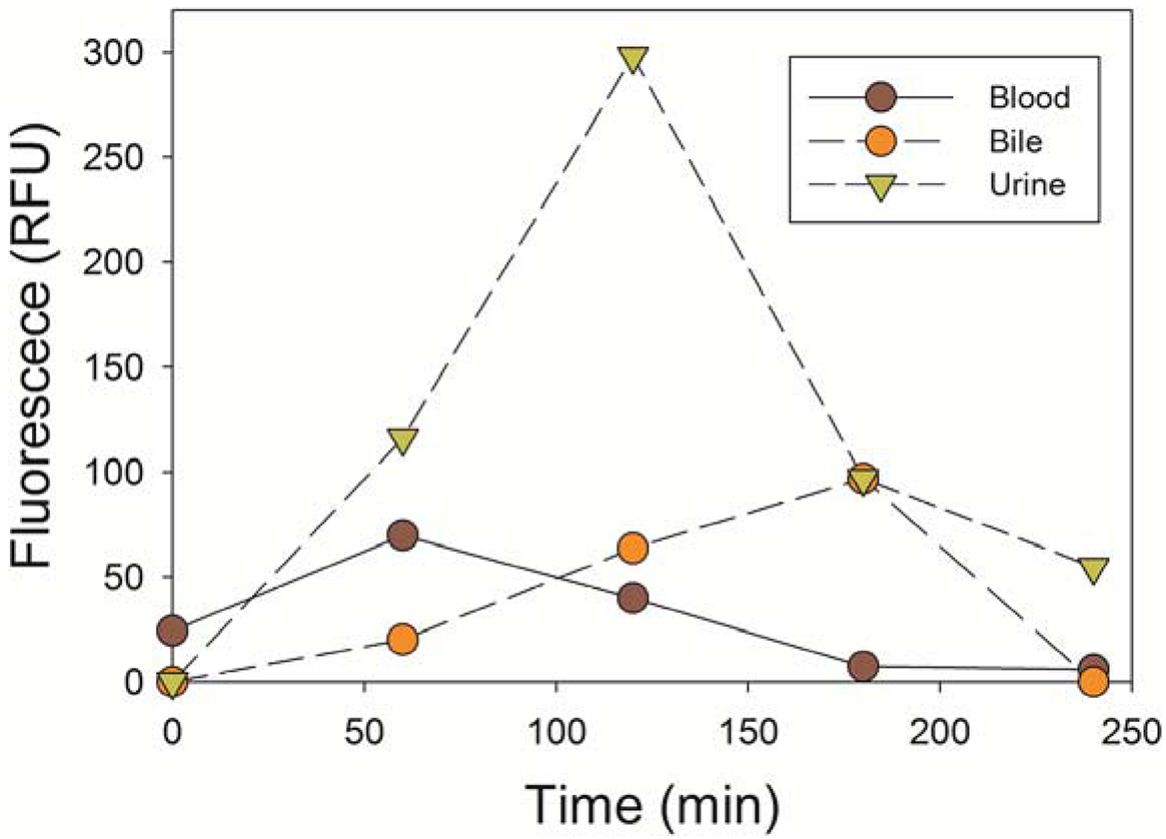
Absorption and clearance of GE3126 in the porcine model. The fluorescence emission intensity of GE3126 was measured in biological fluids collected at indicated time points.

Blood, urine and bile samples were collected before the IV injection of GE3126 and every hour post- injection. To minimize the effect of potential metabolites of GE3126 in blood, bile and urine, we measured the fluorescence intensity at optimal excitation and emission for GE3126 (Fig 7). The peak fluorescence in blood was 1 h after IV injection, with elimination through primary clearance (urine). The majority of the fluorophore was cleared after 4 h.

## Discussion

Unintended injury to peripheral nerves occurs in many lifesaving surgical procedures, and there is currently no system in the clinic for real-time identification of nerves in either the open or laparoscopic surgical settings. The development of fluorescent nerve imaging agents is made more challenging by the specialized physiology of the blood-nerve barrier, which can block the delivery of molecules to the nervous system. An effective imaging agent, in addition to being fluorescent, must have the ability to penetrate this barrier and exhibit high selectivity for a highly abundant nerve target, since it has to diffuse across capillary beds with tight junctions after IV administration or local injection. Molecules that are able to cross the blood-nerve barrier are lipophilic. Because of this, some degree of non-specific fluorescence signal in adipose tissue is to be expected, and every effort must be made to minimize this effect. Additionally, the molecular weight should be less than 700 g/mole, with 400 g/mole as the ideal [39]. Thus, the targeting moiety has to be inherently fluorescent because conjugating it to a dye would significantly increase its molecular weight beyond the desirable range.

Improvement in the aqueous solubility of GE3126 made possible the complete removal of DMSO in the final IV formulation, thus minimizing the potential for formulation-related adverse effects. The results also revealed that scaling by body surface area from small to large animal was acceptable in determining the dose for IV injection. A final dose of 0.74 mg/kg was injected into the porcine model, showing no adverse effects in pigs. This dose was 47 times lower than the dose administered in rats (35 mg/kg) for the non-clinical modified Irwin screen, which showed a lack of observable major adverse events in rats within the duration of the Irwin screen. Physiologic effects at extended time points, if there are any, will be determined in the future.

Nerve visualization was observed rapidly in the porcine model, within 5–10 minutes post-injection and the nerve fluorescence signal was maintained for up to 80 minutes. The fluorescence signal in adipose tissue were attenuated and the muscle remained dark throughout the procedure, demonstrating that the contrast between nerve and surrounding muscle and adipose tissue was much improved in the larger animal, thereby increasing nerve visibility. The decrease in adipose tissue fluorescence could be caused by lower non-specific partitioning to adipose tissue as a result of the increased aqueous solubility of GE3126, or an increase in the clearance rate from adipose tissue. Since the fluorescence properties of GE3126 is sensitive to the solvent environment, similar to other push-pull bis-styryl dyes [33], another potential explanation could be that the quantum yield of GE3126 was different once it was partitioned into the porcine adipose tissue. Another factor to consider is that fluorescence intensity measurements may be complicated by scattering and absorption in different tissue types, so comparison of intensities across different tissue types may not be suitable for determination of relative dye concentration in each type of tissue. Rather than solely relying on measuring fluorescence intensity, future work will include a more quantitative measurement of tissue biodistribution.

Several recent works have established the advantages of contrast agents for fluorescence imaging in the near-infrared (NIR) region, principally because autofluorescence appears predominantly in the visible spectrum, whereas NIR allows for virtually background-free imaging and potentially goes deeper into the tissue [29, 40]. In this work, we demonstrated that intraoperative imaging using a visible dye is also a viable approach under certain conditions. In controlled lighting environments, such as in minimal access surgical procedures, or with tailored operating room lighting for open procedures, a dye with a large Stokes shift can minimize the impact of autofluorescence, similar to NIR imaging, despite operating in the visible spectrum. Moreover, emission in the visible may be preferable, since optical elements in commercial laparoscopes are typically optimized for visible imaging. A potential limitation of imaging in the visible spectrum is that penetration will be limited to near surface (~2–3 mm depth). Nerves can be a challenge to visualize even when they are fully exposed, especially when they are in close proximity to the target pathology, such as the prostate. In some cases, such as in groin and prostate surgery, the fine network of nerves runs in between layers of fascia, with thickness estimated to be ~1–3 mm. GE3126 may improve visualization in cases where nerves are obscured by fascia (such as in Fig 3A and 3B). Outcome studies in surgical animal models will have to be performed to determine the potential of this technology to decrease post-surgical complications resulting from accidental nerve damage.

Other fluorescent nerve contrast agents have been described recently, including non-targeted NIR dyes that are injected directly at the nerve of interest [41], a protein for labeling retrograde transport in nerves [42], and a dye-conjugated peptide that bound to connective tissue in the nerve epineurium and endoneurium [43]. Direct injection to the nerve of interest could require method optimization for each anatomical location. For the dye-conjugated peptide, uptake and fluorescence in fascia, connective tissue, and adipose tissue were observed [44, 45]. Moreover, dye-conjugated peptides are in general more costly to produce and to scale-up compared to small molecule fluorophores, particularly because the adult human dose for the peptide was proposed to be in the range of 1–3 grams (14–43 mg/kg) [43, 44]. A recent report of a nerve-highlighting oxazine dye [44] underscores the critical need of exploring different strategies for nerve labeling. Absorbing and emitting in the orange-red visible region, the dye’s Stokes shift was ~17 nm, which makes efficient separation of excitation and emission difficult, typically at the expense of signal. In porcine imaging, the oxazine dye exhibited lower adipose tissue fluorescence, however its optimal nerve-to-muscle ratio was 4 times lower than GE3126 [44].

In this work, we demonstrated that GE3126 had most of the desired characteristics for fluorescence image-guided surgery, and it had improved properties over our earlier bis-styryl nerve-highlighting contrast agents. In order to simulate the utility of the instrument in a clinical environment, intraoperative imaging was demonstrated using the laparoscopic instrument in a large animal model. This dual-mode instrument uses time-multiplexed illumination and detection for near simultaneous display of white light and fluorescence images. By using a single camera, it allows for complete registration of the images in both modes, and is compatible with any standard laparoscope [34, 45]. One limitation of myelin-targeting fluorophores such as GE3126 is that while one can design the molecules to be less lipophilic, some degree of uptake in adipose tissue will necessarily occur. Hence, novel strategies to suppress or differentiate the fluorescence from adipose tissue may be required. Since the push-pull bis-styryl dyes typically demonstrate spectral sensitivity to the environment (Fig 2C and 2D, Table 2), our future work includes developing approaches that utilize enhancements to imaging instruments and software to suppress adipose tissue fluorescence coupled with further dosing optimization of GE3126 in porcine studies.

## Supporting Information

S1 File Synthesis, Purification and Characterization of GE3126. (PDF)
