# Supplementary material for: Improved Intraoperative Visualization of Nerves through a Myelin-Binding Fluorophore and Dual-Mode Laparoscopic Imaging
Source: PLoS One. 2015 Jun 15;10(6):e0130276. doi: 10.1371/journal.pone.0130276 (PMC4468247; doi:10.1371/journal.pone.0130276)

S1 File. Synthesis, Purification, and Characterization of GE3126

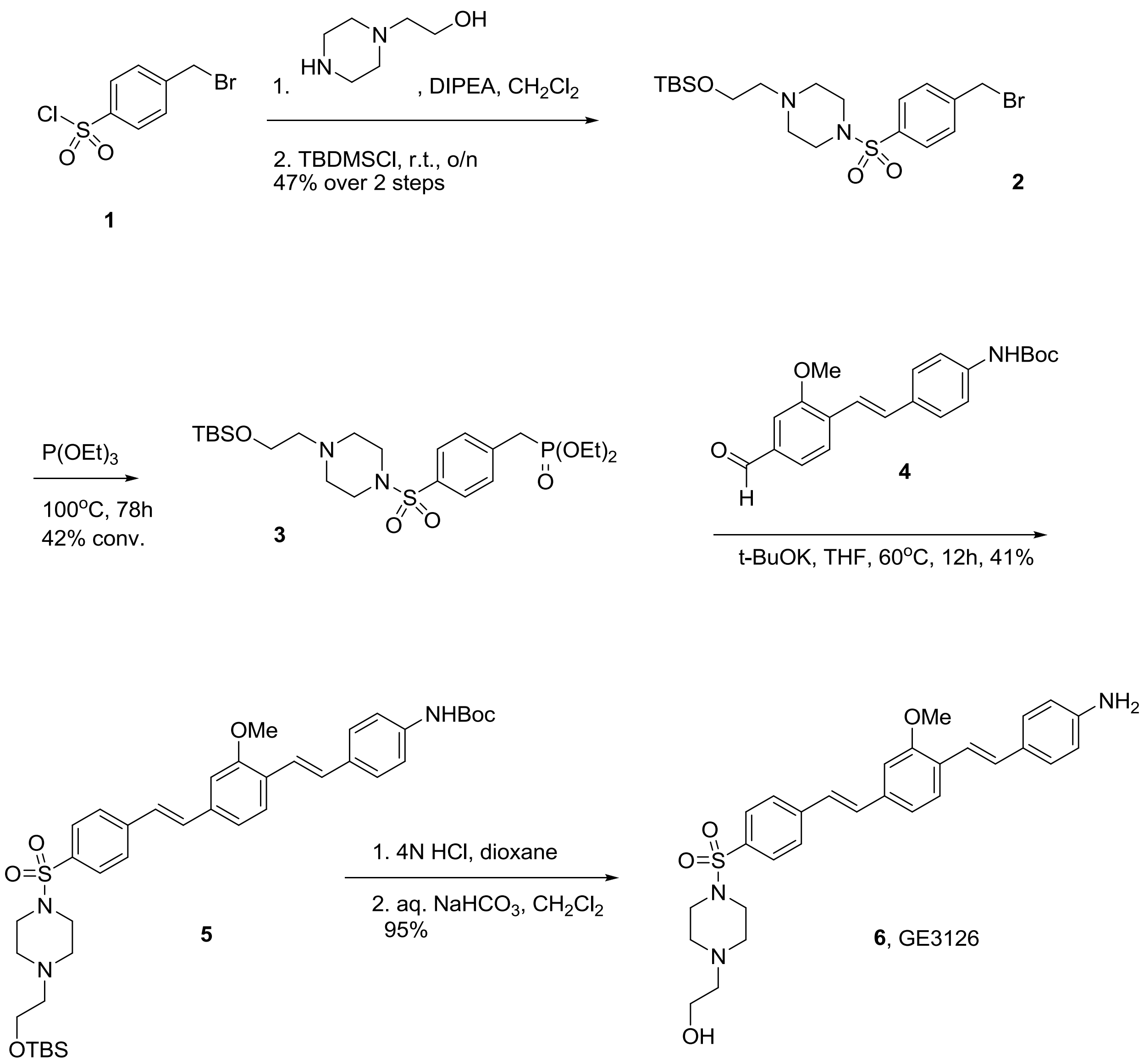

Abbreviations: Diisopropylethylamine (DIPEA), tert-butyldimethylsilylchloride (TBDMSCI), tert-butyldimethylsilyloxy (TBSO), potassium tert-butoxide (tBuOK), tetrahydrofuran (THF), tert-butyloxycarbonyl (Boc), room temperature (r.t.), overnight (o/n), conversion (conv.)

Synthesis of (2): 1-(4-(Bromomethyl)phenylsulfonyl)-4-(2-(*tert*-butyldimethylsilyloxy)ethyl)piperazine

To a solution of 4-bromomethylbenzenesulfonyl chloride **1** (6 g, 22.3 mmol) in anhydrous CH_2Cl_2 (60 mL) at 0°C was added Hunig's base (15.4 mL, 90.8 mmol) followed by the addition of a solution of 1-(2-hydroxyethyl)piperazine (2.5 mL, 20.6 mmol) in anhydrous CH_2Cl_2 (10 mL). The reaction was stirred for 5 h, and then treated with tert-butyldimethylchlorosilane (3.48 g, 23.2 mmol). The reaction mixture was allowed to slowly warm up to room temperature while being stirred overnight, then washed with water (3 times), brine (1 time), then dried over Na_2SO_4, and filtered. The solvents were removed under reduced pressure, and the residue was purified on a 120 g silica gel column (eluent: hexane/ethyl acetate 0%-25% gradient) to give **2** (5 g, 51% yield). ^{1}H NMR (CD_2Cl_2) δ ppm: 7.77 (d, J = 8.4 Hz, 2H), 7.62 (d, J = 8.4 Hz, 2H), 4.69 (s, 2H), 3.68 (t, J = 6.0 Hz, 2H), 3.04-3.00 (m, 4H), 2.63-2.59 (m, 4H), 2.51 (t, J = 6.0 Hz, 2H), 0.89 (s, 9H), 0.04 (s, 6H).

Synthesis of (3): Diethyl 4-(4-(2-(tert-butyldimethylsilyloxy)ethyl)piperazin-1-ylsulfonyl)benzylphosphonate

Compound **2** (590 mg, 1.24 mmol) was dissolved in triethyl phosphite (1.69 mL, 9.88 mmol) and the mixture was stirred at 100°C for 78 h while being monitored by LC/MS. After the complete conversion of the bromide, the excess triethyl phosphite was removed on a rotary evaporator. The resulting residue was purified on a 12-g silica gel column (eluent: hexane to 30% ethyl acetate in hexane) to yield **3** (352 mg, 53% yield). ^{1}H NMR (CD_2Cl_2) δ ppm: 7.71 (d, J = 8.4 Hz, 2H), 7.52 (dd, J_1 = 8.4 Hz, J_2 = 2.4 Hz, 2H), 4.16-4.00 (m, 4H), 3.68 (t, J = 6.0 Hz,

2H), 3.24 (d, J = 22.0 Hz, 2H), 3.07-2.98 (m, 4H), 2.63-2.58 (m, 4H), 2.50 (t, J = 6.0 Hz, 2H), 1.27 (t, J = 7.2 Hz, 6H), 0.89 (s, 9H), 0.04 (s, 6H). MS (ESI+): 536 $(M+H)^+$.

Synthesis of (5): *tert*-Butyl 4-(4-(4-(4-(2-(tert-butyldimethylsilyloxy)ethyl)piperazin-1-ylsulfonyl)styryl)-2-methoxystyryl)phenylcarbamate

To a dry vial containing compound **3** (1.13 g, 2.1 mmol) under N_2 was added dry tetrahydrofuran (THF) (10 mL) followed by the dropwise addition of a solution of potassium *tert*-butoxide (350 mg, 3.13 mmol) in dry THF (3.0 mL) at 0 °C. The ice bath was removed after 0.5 h, and the reaction mixture was allowed to stir at room temperature for another 25 min. At this point, a solution of (E)-*tert*-butyl 4-(4-formyl-2-methoxystyryl)phenylcarbamate **4** (707 mg, 2.0 mmol) in dry THF (5 mL) was added dropwise at 0 °C, and the mixture was warmed and stirred at 60 °C overnight. The reaction mixture was then partitioned between ethyl acetate (250 mL) and water (200 mL). The aqueous layer was extracted with additional ethyl acetate (2x 200 mL) and washed with water (150 mL), then dried over $MgSO_4$. The solvent was evaporated under reduced pressure and the remaining crude product was purified on a 40 g silica gel column (eluent CH_2Cl_2 to 5% methanol in CH_2Cl_2) to yield the desired product **5** as a yellow solid (648 mg, 51% yield). ^{1}H NMR (CD_2Cl_2) δ ppm: 7.80-7.70 (m, 4H), 7.64 (d, J = 8.0 Hz, 1H), 7.52 (d, J = 8.6 Hz, 2H), 7.47-7.39 (m, 3H), 7.33-7.12 (m, 5H), 6.86 (s, 1H), 3.99 (s, 3H), 3.72 (t, J = 5.8 Hz, 2H), 3.20-3.00 (m, 4H), 2.75-2.64 (m, 4H), 2.55 (t, J = 5.8 Hz, 2H), 1.56 (s, 9H), 0.90 (s, 9H), 0.06 (s, 6H). MS (ESI+): 734 $(M+H)^+$, 756 $(M+Na)^+$.

Synthesis of 6 (GE3126): 2-(4-(4-(4-(4-Aminostyryl)-3-methoxystyryl)phenylsulfonyl)piperazin-1-yl)ethanol hydrochloride

To a solution of compound **5** (570 mg, 0.78 mmol) in 1,4-dioxane was added slowly 4 *N* HCl/dioxane (8 mL) at 0°C. After 30 min, the cold bath was removed and the reaction mixture was stirred at room temperature for 4 h. After completion of the reaction, the solvent was evaporated, and the remaining viscous residue was triturated with hexane (150 mL) and diethyl ether (150 x 2 times) in order to remove the *tert*-butyldimethylsilanol. The crude product was dissolved in CH_2Cl_2 (120 mL), and neutralized by stirring with a saturated solution of $NaHCO_3$ (100 mL) for 30 min at room temperature. The organic layer was separated and the aqueous layer was extracted with CH_2Cl_2 (200 mL); the combined organic phase was dried over Na_2SO_4 to give 400 mg of free base.

Purification by reverse phase-MPLC: 150 mg of the yellow free base product obtained as above was purified by MPLC (ISCO) on a reverse phase column (30 g, C18) using water-acetonitrile gradient 0-70%, to give 50 mg of pure product (>99% by LC with fluorescent detection).

Purification by reverse phase-HPLC: The scaled-up reaction product was purified on a pre-packed Waters Symmetry C18 (250 x 50 mm, 7 μm) on a Delta Prep 2000/4000 system equipped with #7 pump heads and a Waters 484 UV-Vis detector. A scout run of 250 mg was dissolved with sonication in a 1:1 mixture of water: MeOH containing 0.1% v/v TFA. The purification was run isocratically at 50:50 v/v water: MeOH containing 0.1% v/v TFA, a flow rate of 80 mL/min, and detection at 375 nm. Following elution of GE3126 the column was flushed with acetonitrile containing 0.1% v/v TFA. The sample load was incrementally increased to 500 mg/ run. A total of 8 g GE3126 was purified using this method.

The fractions containing purified GE3126 were pooled and concentrated by rotary evaporation. The residual TFA was removed by solid phase extraction (SPE) prior to lyophilization. The aqueous solution of GE3126 was loaded on a Symmetry C18 (250 x 50 mm, 7 μm) column previously equilibrated in 5% MeOH: water. Upon water washing, the compound was eluted in a narrow band with acetonitrile:water 95:5 v/v. The eluate was concentrated to about 10% of its original volume and the residue was lyophilized to give the pure GE3126 dye as a fluffy red-brown solid.). MS (ESI+): 520 $(M+H)^+$. NMR spectroscopy data for GE3126 (20 mg/mL in DMSO-d_6, Bruker 600MHz) are shown below.

^{1}H NMR (DMSO-d6, 600 MHz) δ ppm: 13.2 (brs, 3H), 7.87 (d, J=12 Hz, 2H), 7.78 (d, J=12 Hz, 2H), 7.72 (d, J=12.6 Hz, 1H), 7.68 (d, J=11.4 Hz, 2H), 7.48 (d, J=8.4 Hz, 2H), 7.44 (d, J=18 Hz, 1H), 7.37 (d, J=12 Hz, 2H), 7.34 (s, 1H), 7.3 (d J=18 Hz, 1H), 7.28 (d, J=12.6 Hz, 1H), 3.96 (s, 3H), 3.73 (d, J=15.6 Hz, 2H), 3.68 (t, J= 3.6 Hz, 2H), 3.52 (d, J=15.6 Hz, 2H), 3.12-3.28 (m, 4H), 2.74 (t, J=7.4 Hz, 2H).

^{13}C-NMR (DMSO-d6, 600 MHz): δ 158.67, 158.41, 158.15, 157.90, 156.95, 142.49, 137.55, 132.34, 132.18, 128.37, 127.89, 127.59, 127.25, 126.95, 126.83, 125.56, 123.82, 123.70, 120.01, 117.94, 116.03, 114.12, 112.20, 109.66, 57.55, 55.73, 54.76, 50.48, 42.94.

^{1}H and ^{13}C GE3126 NMR assignments:

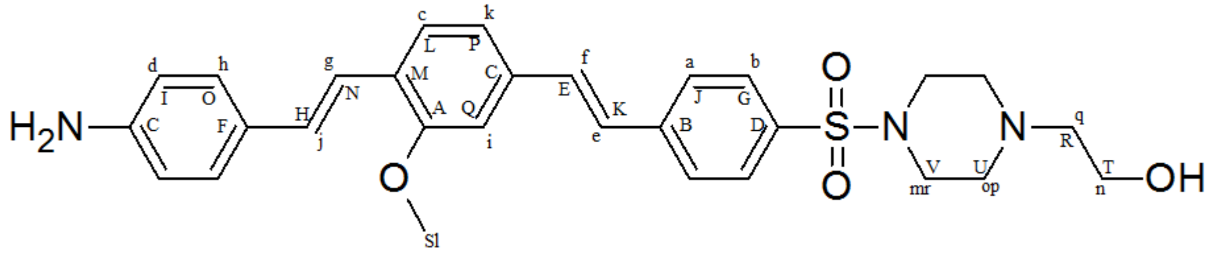

GE3126 ^{1}H-^{13}C labels:

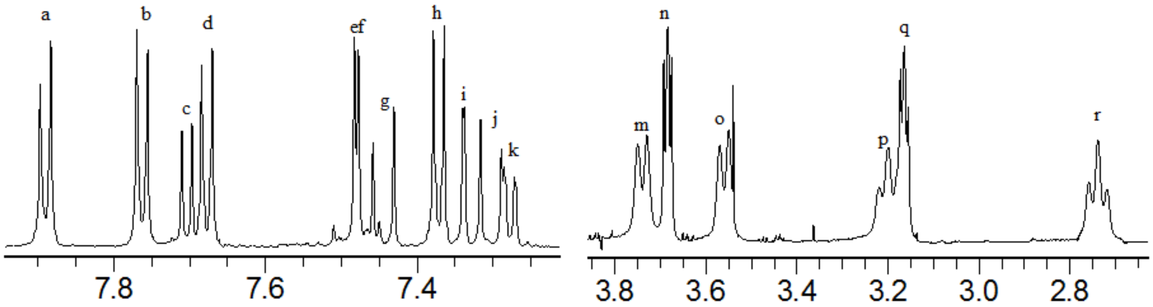

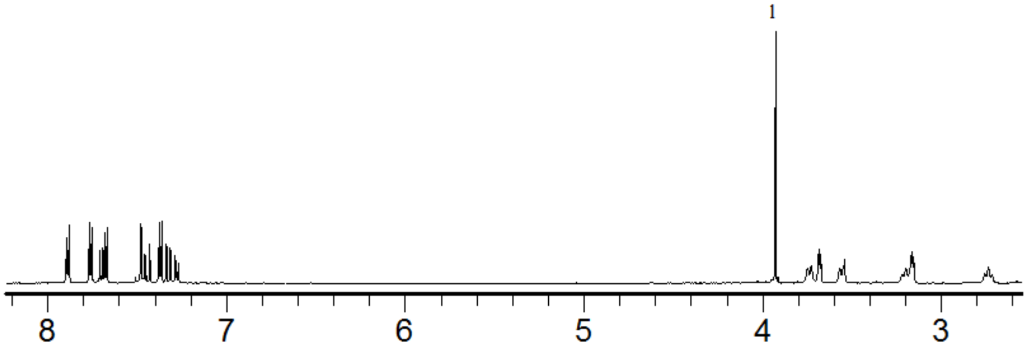

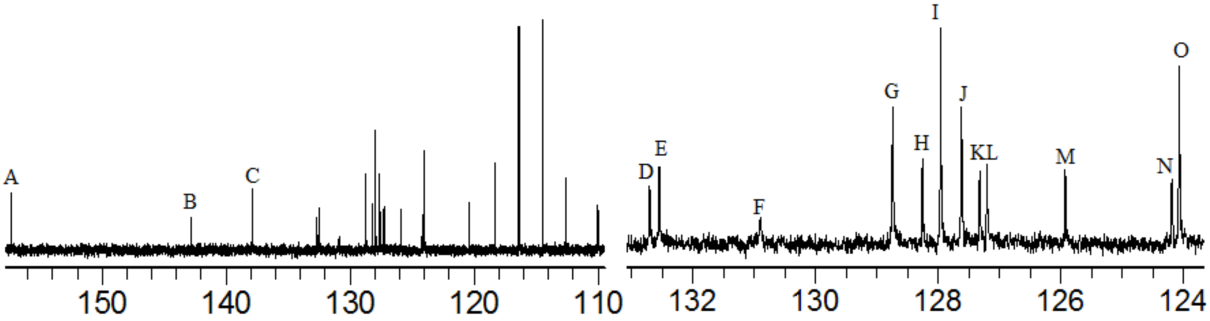

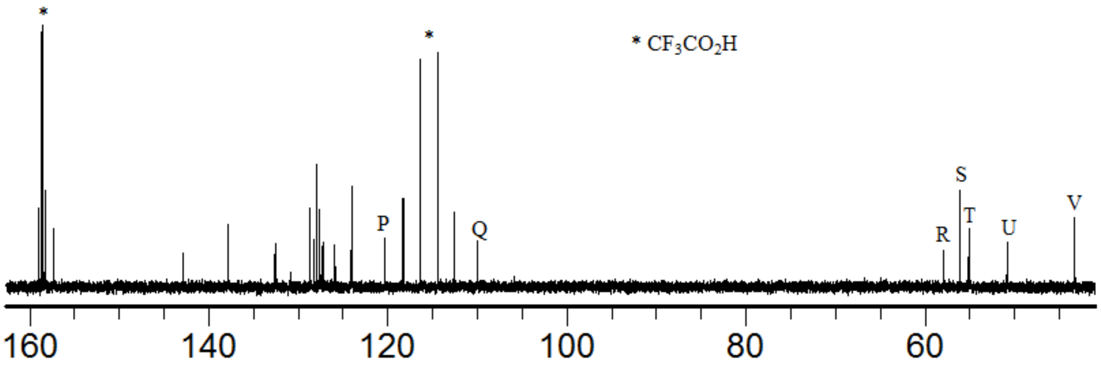

GE3126 ^{13}C NMR (Standard, top) and attached proton test NMR (APT, bottom):

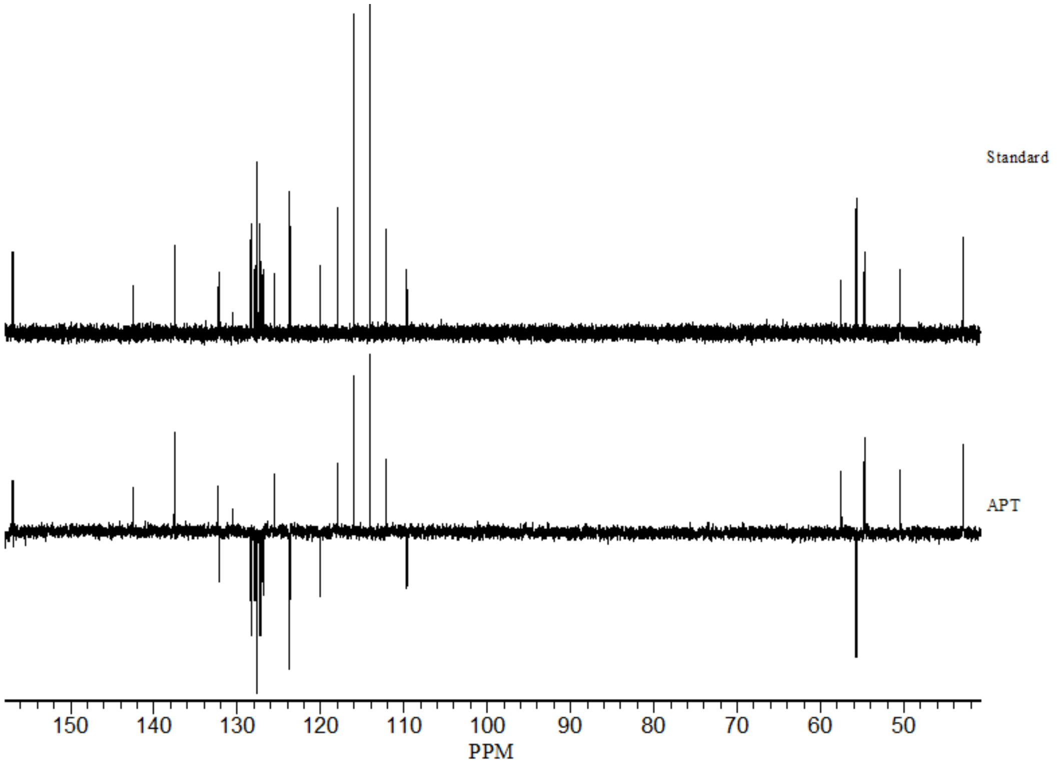

Correlated NMR spectroscopy (gradient heteronuclear single quantum correlation-gradient heteronuclear multiple bond correlation, gHSQC-gHMBC). Right figure is expanded region from the left figure:

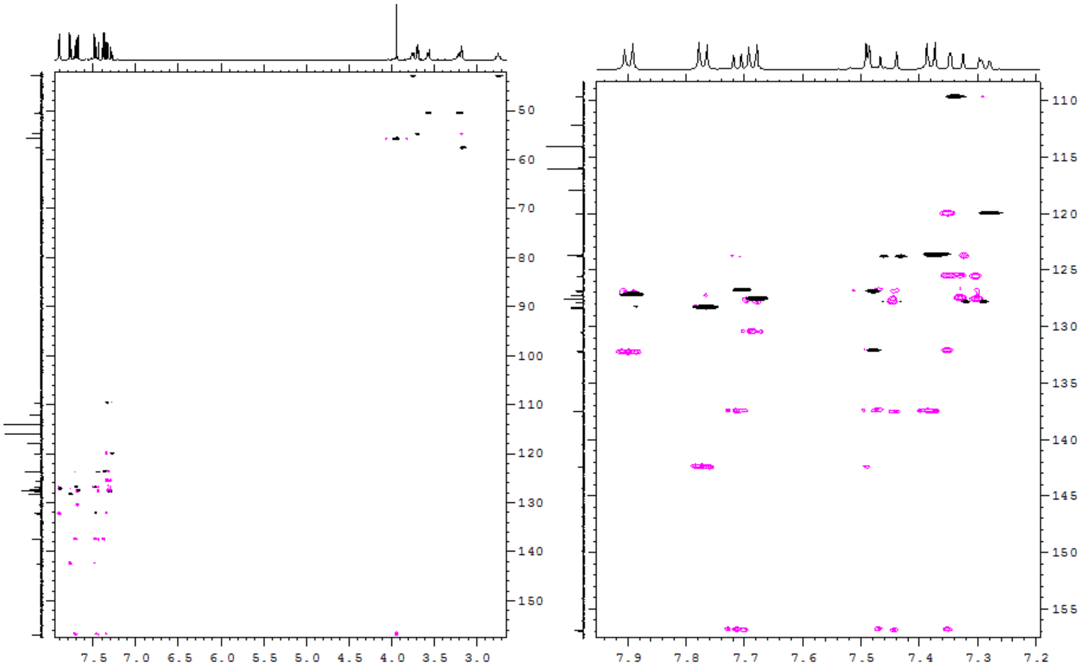

GE3126 gradient correlation spectroscopy (gCOSY):

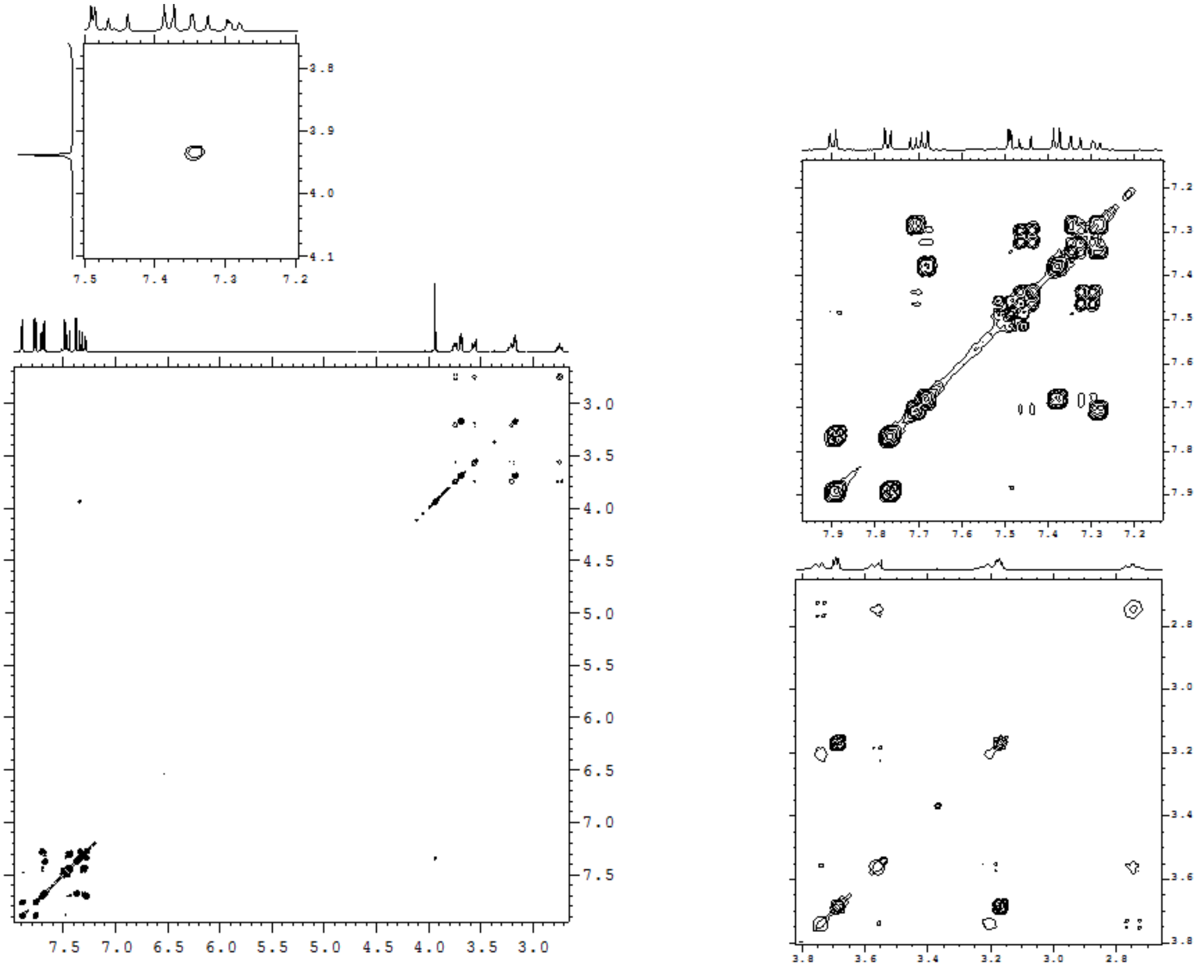

Supplement: S1 File — (PDF) [file pone.0130276.s001.pdf]
